# A lifeworld theory-led action research process for humanizing services: improving “what matters” to older people to enhance humanly sensitive care

**DOI:** 10.1080/17482631.2020.1817275

**Published:** 2020-11-23

**Authors:** Kathleen T. Galvin, Carole Pound, Fiona Cowdell, Caroline Ellis-Hill, Claire Sloan, Sheila Brooks, Steven J. Ersser

**Affiliations:** a School of Health Sciences, University of Brighton, Brighton, UK; b Faculty of Health and Social Sciences, Bournemouth University, Bournemouth, UK; c Faculty of Health, Education and Life Sciences, Birmingham City University, Bournemouth, UK; d Department of Health Sciences, Faculty of Sciences, University of York, Heslington, UK; e Bournemouth University, Bournemouth, UK

**Keywords:** Humanized care, lifeworld-led care, phenomenology, service improvement, action research, skin care, stroke rehabilitation, care

## Abstract

**Purpose**: Using a theory-led action research process test applicability of humanizing care theory to better understand what matters to people and assess how the process can improve human dimensions of health care services. Consideration of the value of this process to guide enhancements in humanly sensitive care and investigate transferable benefits of the participatory strategy for improving human dimensions of health care services.

**Methods**: Action research with service users, practitioners and academics, with participatory processes led through the application of theory via a novel Humanizing Care Framework in two diverse clinical settings.

**Results**: Participants engaged in a theory-led participatory process, understood and valued the framework seeing how it relates to own experiences. Comparative analysis of settings identified transferable processes with potential to enhance human dimensions of care more generally. We offer transferable strategy with contextualized practical details of humanizing processes and outcomes that can contribute to portable pathways to enhance dignity in care through application of humanizing care theory in practice.

**Conclusion**: The theoretical framework is a feasible and effective guide to enhance human dimensions of care. Our rigorous participative process facilitates sharing of patient and staff experience, sensitizing practitioners’ understandings and helping develop new ways of providing theoretically robust person-centred care based on lifeworld approaches.

## Introduction and background

Patients and people who use health services indicate that they do not always feel met as human persons in the way that care is organized and practised. Literature points to the challenges of delivering humanly focused care and significant care failings (Department of Health and NHS Commissioning Board, 2012; Francis, 2013; Sabo, 2006). In the context of this present study, in dermatology and stroke rehabilitation settings a detailed picture of how personhood is easily obscured is apparent. For example, in dermatology, health care staff are inclined to treat patients with an emphasis on their skin condition alone, rather than as a whole person (Nguyen et al., 2013; Tan et al., 2016) and despite increasing knowledge about the need for more human-focused care this problem persists over time (Chisholm et al., 2016). This tendency to treat the skin disease rather than the person who lives with a skin condition is an example of a reductionist view of the body obscuring other human dimensions of care. Despite significant differences in population and health services offered, similar themes are evident within care practices in the experience of stroke care literature.

A recent meta-synthesis of the experience of stroke rehabilitation services concludes that there needs to be an equal focus on social and psychological dimensions as well as the physical in order to ensure dignified care. Services need to be expanded to help a person focus on their recovery in their unique social world (Reed et al., 2012). Although outcomes for stroke survivors have improved greatly (Morris et al., 2019), patients and their carers still ask for more individualized approaches to care that are person centred. There is a significant call for consideration of the whole person in the context of their rehabilitation (Hole et al., 2014) a more balanced emphasis, beyond physical needs alone, with attention to the social, emotional and psychological impacts of stroke (Arntzen & Hamran, 2016,) and have highlighted how difficult this is to achieve on a stroke unit (Ryan et al., 2017). Literature from both skin health care and stroke rehabilitation clearly points to the scale needed for more attention to the human dimensions of care.

Lifetime prevalence of common skin disease among a random sample of the general European population (n = 12,377) aged 18–74 years showed a lifetime prevalence of, contact dermatitis (15%), atopic dermatitis (7.9%), other eczema (14.2%), psoriasis (5.2%) and skin cancer (2.6%) Svensson et al. (2018). In the UK skin disorders are extremely common, for instance, in England they affect more than half the population each year. Currently this leads to around 13 million primary care consultations and 880,000 referrals to dermatology outpatient clinics (NHS England (2019) Transforming elective care services). People with conditions such as psoriasis and malignant and non-malignant skin lesions (frequent causes of referral to secondary care) often face physical and psychological challenges that adversely affect quality of life and social functioning (Tuckman, 2017). Psoriasis can lead to negative body image and relationship problems (Gündüz et al., 2020) and anxiety, stress, depression are common (Kwan et al., 2018). There is considerable evidence that individuals may face stigmatization and social rejection as a result of living with such a visible condition (Alpsoy et al., 2017; Jankowiak et al., 2020). Treatment satisfaction and experience of dermatology consultations are often poor for people with psoriasis (Van Cranenburgh et al., 2013) against a backdrop for instance, that malignant and non-malignant skin lesions carry a high psychological burden Tavakolpour et al., 2017) and we already know that needs for supportive care are often unmet (Körner et al., 2016).

The picture is similar for stroke, also an example of patient needs of large scale, there are more than 100,000 new strokes in the UK each year and over 1.2 million stroke survivors in the UK (Stroke Association, 2019). Ageing populations and increased survival rates from stroke indicate a significant increase in the burden of stroke across European countries and this has implications for the quality of person-centred care (Stevens et al., 2017). For instance, evidence suggests that emotional and existential needs of stroke survivors and their family are largely unmet, anxiety, depression are commonplace and people feel abandoned (Stroke Association, UK, 2013). An ethnographic study of stroke units in the UK suggested human factors and quality of care and rehabilitation can be overlooked as units strive to meet stroke audit targets (Taylor et al., 2018). The perspective of patients, family and care providers indicates that quality of personal focused care and patient-staff relationships in stroke care can be undermined by pressure to meet targets and discharge patients (Ryan et al., 2017). In this context, Lawton et al. (2016) have reviewed the importance of therapeutic alliance between stroke survivors and professionals and the building of trusting relationships (Luker et al., 2015).

Use of a novel theoretical framework delineating dimensions that constitute a feeling of being human or feeling dehumanized, we believe offers a practical step forward. For example, consideration of dimensions that constitute a feeling of being human may deepen practical directions from the six espoused values of Care, Compassion, Courage, Communication, Competence and Commitment, “the 6 C’s” (Department of Health and NHS Commissioning Board, 2012). The 6 C’s build on previous phenomenological work, Roach (2002) theorized professional caring values and outlined attributes for caring in a Canadian study. These concepts were developed further in a vision and strategy by the UK (UK) Chief Nursing Officer, who outlined a strategy for building a culture of compassionate care based on these six values (Department of Health and NHS Commissioning Board, 2012) within UK National Health Service (NHS). Similarly, there have been policy moves in other European countries to enhance patient- led or person-centred care. Against this current policy backdrop, we are attempting to take a foundational step back, returning to what matters to older people in care and clinical settings by understandings that come directly from “the lifeworld”. The lifeworld for the purposes of this study refers to a particular view of the person as humanly living in the seamlessness of everyday life that includes the following experiential dimensions for the person receiving care: temporality (experience of time), spatiality (experience of space), embodiment (experience as this body), sociality, (or being in relation to others) (see full discussion in the context of lifeworld approaches to care for example, Dahlberg et al., 2009; Galvin & Todres, 2013). An entry point for practical actions to enhance humanly sensitive care can be achieved by attending to experiences of “what it is like” for the older person, sensitized by a theoretical framework that focuses on what makes them feel more human or less human in that context. This participatory research study is one attempt to examine the usefulness of this approach and is one that is allied to a range of arts-based knowledge mobilization approaches within health services (Scott, 2013).

### Rationale: “lifeworld-led care” through humanizing approaches

We advocate an approach to care that is founded on a phenomenological, lifeworld-led approach (Dahlberg et al., 2009; Todres, 2007). While ideas about the lifeworld are not new, there is a case to be made for how such phenomenologically oriented ideas can be used to inform practical directions in care settings. The humanization theoretical framework, informed by the lifeworld (Todres et al., 2009) comprises eight dimensions of humanization and dehumanization that have been subsequently delineated and demonstrated as useful in practice application (Galvin et al., 2018; Borbasi et al., 2012). These *do not* form a checklist, nor are they prescribed generalizations. Instead, the eight bipolar dimensions, are points of emphasis, that delineate what can make a person feel “more” or “less” human.

**Figure 1.**
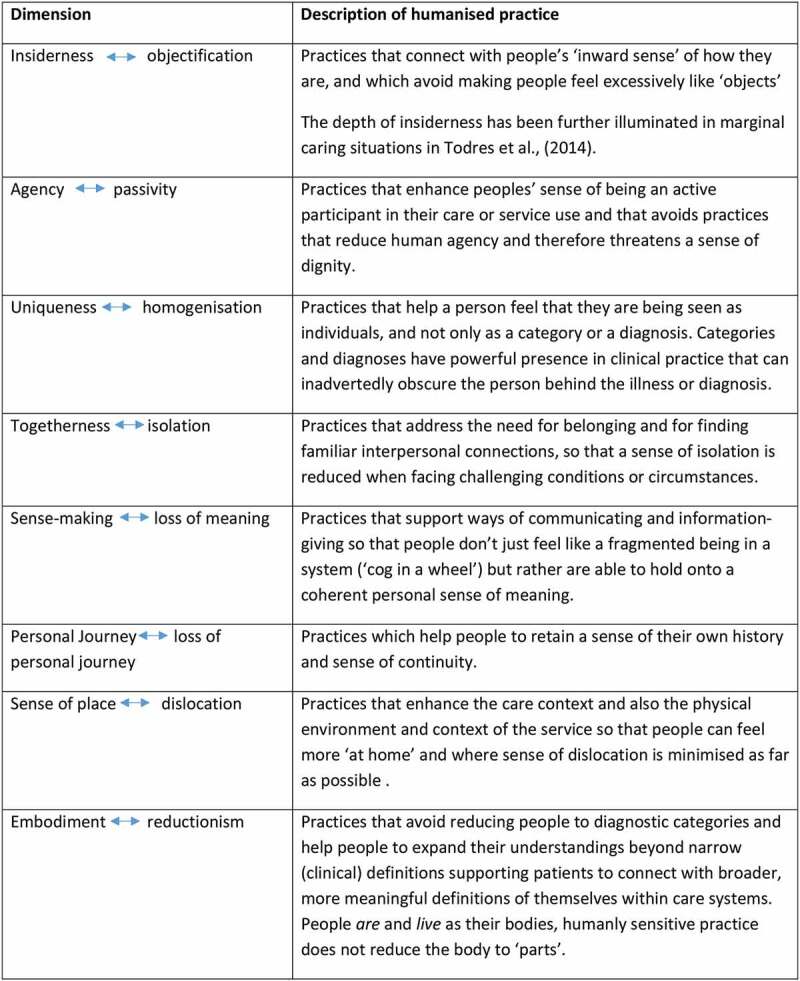
The eight dimensions of humanizing care (after Todres et al., 2009)

Figure 1 below summarizes these eight human dimensions of care, each with their commensurate form of dehumanization as an emphasis. Together, these emphases delineate aspects of what it is to be and feel human and can also point to what needs to be attended to in meeting needs as human persons within care settings. Conversely, forms of dehumanization present threats to experiencing a situation as a human person. For example, a sense of feeling human can be inadvertently obscured if there is an undue overemphasis on the technical and organizational aspects of care, thereby undermining care responses that are humanly sensitive. We acknowledge that a necessary emphasis on technical aspects of care is sometimes required in acute and critical situations, and sometimes patients are comfortable handing themselves over for necessary technical care that is instrumental, however, the obscuring of human aspects of care becomes a problem negatively impacting patients if the mode of care becomes stuck in only the technical aspects, particularly for example, in long-term conditions. The human dimensions of care are easily obscured and can also get lost or dropped out altogether in these situations if they are not actively attended to. It is important to note that each dimension is considered as an emphasis along a continuum, they are not binary opposites but rather, they are all intertwined, acting together as a background, but where different emphases can stand out and have relevance in different situations. Figure 1 provides a summary. For further detail regarding the nature of these dimensions and how they were developed drawing on a phenomenological orientation, readers are referred to Todres et al. (2009).

For the purposes of this paper our aim is to offer a rigorous practical direction to respond to current health care policy that focuses on enhancing patient experience. In this regard, healthcare professionals need a transferable process that illuminates understandings, concerns and experiences of older adults and which has its foundation in their lifeworld. The dimensions summarized in Figure 1 could be used as a sensitizing background to help practitioners attend to and enhance humanly sensitive healthcare practice through a form of attunement to what it feels like to be human and what it feels like to be dehumanized. Therefore, for the purposes of a service improvement project, our focus was to draw attention to how services were experienced by older people, specifically by exploring and then attending to the eight humanizing dimensions of care as directions for practice. The participatory process included a testing out of the usefulness of application of the humanizing dimensions. This present paper focuses on the applicability of the humanized care theoretical framework and the transferable aspects of a novel theory-led action research strategy that was used. Tripartite action research groups composed of older service users, a range of healthcare professionals (including nurses, therapists and healthcare assistants) and academics, met in two purposively selected diverse care settings, a dermatology out-patient clinic and a stroke rehabilitation unit to consider the human dimensions of care and assess theory pplicability to practice improvements in each setting.

## Research aim and objectives

The overall aims were to:

1. Use a humanizing theoretical framework to contribute to better understanding of what matters to older people in collaboration with them.
2. Explore the use of these insights to enhance humanly sensitive care.
3. Investigate the extent to which the benefits of theory-led action research strategy, sensitized by new theory for improving the human dimensions of care were transferable to other settings and services.

### Objectives

The objectives of each theory-led action research group (ARGs) were to:

1. Introduce the theoretical framework based on humanized care and explore how older people engage with the humanizing dimensions
2. Investigate what experiences and practices are important to older people in making them feel human, using the theory as a guide
3. Identify the human aspects of health care practice that could be developed within a dermatology outpatient clinic and a stroke rehabilitation unit
4. Identify transferable processes with potential to enhance care for older people in other human service settings.

## Method

### Study design

Action research methodology, sensitized and led by lifeworld theory (“experimental action research” categorized by Hart & Bond, 1996), was used to: (a) Achieve a participatory form of patient led reflection with discussion of any “humanizing”and “dehumanizing” aspects of care (b) Facilitate decision-making on what kind of humanized care changes could be achieved (c) Reflect on what impacts findings might have on the care of older people in specialist hospital care settings. It was anticipated that such a theory-led action research approach would provide a strong basis for sustaining any changes implemented beyond the life of the project. Our approach focused on participatory principles with introduction and sensitization to the humanizing care conceptual work, reflecting experimental action research (Hart & Bond, 1996). Experimental action research has the following features: the problem focus is introduced by the researcher (in this case the need for attention to the human dimensions of care); there is an interaction of social science theory with practical social problems (in this case novel humanization of care theory with how aspects of the care service are experienced by service users); and evaluation of the outcomes which tends to be more researcher led, though in practice there is often a shift along the continuum of the action research typology, becoming more participatory and empowering as the project unfolds (in this case a tripartite group of service users, professionals in the setting and researchers worked together as an action research group and demonstrated a high degree of mutual participation).

### Research governance and ethical approval

Ethical and research governance approval was secured from the Faculty of Health and Social Care, University of Hull, and the Proportionate Review Sub-Committee of the NRES Committee North East—Sunderland (REC Reference: 14/NE/1046; IRAS project ID: 150621) and both NHS sites.

Settings

The inclusion of participants with differing health conditions in two contrasting care settings enabled the academic team to assess what aspects of humanized theory application are most transferable and what aspects are most important to older patients and service users. Two geographically distinct sites were chosen, one in southern and one in northern England. Both settings are high pressure clinical environments that operate in complex environments of change, policy drivers, local NHS and UK national imperatives which are relevant internationally. There are a number of similarities in the context of both settings that are important to draw out as a background for participatory project work that engages participants in enhancing humanized care. These include the nature of the specialist settings for older people which includes a high level of expertise constituted by clinical teams. It is an important feature of the project that the application of the humanizing framework was attempted in typical conditions for each setting to aid transferability, ensuring that the global characteristics of both settings that are similar and different noted. Key differences between the two research sites are summarized in Table I.

**Table I.** Study context: Summary of key service differences across both study sites

Dermatology outpatient service (North of England)	Stroke rehabilitation service (South of England)
**Health Care Condition Characteristics and impact on service users**	
Condition requires access via GPs with some delays and gatekeeping Most service user ARG members have lived with skin condition for many years Illness trajectory typically long-term condition with treatment, improvement, periodic flare ups All service users in the ARG still in contact with service Typically service users are ambulant and independent	Condition requires rapid access to service typically via emergency route Most service user ARG members have only recently experienced stroke (months-years) Illness trajectory typically one off acute event followed by rehabilitation and re-enablement. All service users in the ARG now discharged from service Many potential service users unable or unwilling to participate in ARG due to ongoing complex physical, cognitive, communication issues or transport difficulties
**Service provider Characteristics**	
Typically providers in the ARG have had long-term contact with ARG service users (up to 40 years) Less diverse mix of staff members in unit and ARG ARG members tend to be more mature (two semi-retired) and have worked on unit for many years (max range 25 years)	Typically providers in ARG have had minimal or no contact with service users in ARG (days-weeks) More diverse multi-disciplinary staff mix in unit and ARG ARG members tend to come from younger age group and have worked for less time on unit (1 − 13 years range)
**Clinical setting characteristics**	
Out-patient service offering long-term access and re-referral More emphasis on nursing and medical care—greater sense of medical dominance Perception from staff and service users that dermatology is viewed differently to acute care Nursing leadership in unit undergoing staff change Has a research nurse leading mostly clinical trials.	In-patient unit with short term community support through a two- week support service Multi-disciplinary staffing on the stroke unit. Stroke Unit recognized as a beacon within other older people services in the Trust Stable nursing leadership in unit and strong support for project Strong research culture on unit with multiple research projects and clinical trials
**Action Research Group processes**	
Two hour session timed to co-ordinate with staff lunch sessions and clinic times Service users very consistent in attendance but committed staff participants need to work hard to juggle rotas and leave to attend Explicit process used to introduce humanizing dimensions A more verbal presentation of dimensions and educational style in weeks one-four Use of large group process	90 minute session timed to account for service user fatigue and post lunch time staff handover Service users and providers consistent in attendance though one staff member stopped attending after week four Implicit process used to introduce humanizing dimensions A more participatory process with use of creative materials in weeks one-four Mix of small and large group work

### Participants

The number of patients/service users were chosen to ensure that people receiving services did not feel “outnumbered” by staff members. The size of the group, 10 to 12, was consistent with best practice in facilitating action research groups (Bradbury, 2015). Within our research process we were specifically focused on primacy to participants’ experiences of using each contrasting service, all the service users from the stroke rehabilitation service were patients admitted to the in-patient ward following acute cerebral vascular accident and who used the rehabilitation in-patient service until their discharge from hospital, whereas the service users in the dermatology setting were out patients who attended the dermatology outpatients on a regular basis for treatment/follow up. Both groups of service users were contrasting according to setting of services they drew on for their needs, but within each setting the group were all similar in needs and in their use of the typical care of each respective service setting for their long-term condition. Purposive sampling was employed alongside the inclusion and exclusion criteria for selecting participants (Gentles et al., 2015).

### Inclusion criteria (service users)

1. Aged ≥ 65 years
2. Medically stable
3. Able to participate in group conversation
4. Able to attend meetings

### Inclusion criteria (practitioners)

1. Currently working in or familiar with the clinical setting
2. Able to attend meetings within working hours.

### Recruitment and retention

Recruitment was undertaken via informal discussions, an “advertisement” and an email invitation to staff. Staff members made initial contact with patients and service users, if interested academics made telephone contact. All participants received an information sheet prior to taking part. We invited potential participants to attend a question and answer session to learn more about the project and the proposed activities. This served as an important taster session and confidence builder and was a deciding feature for some. Reasons for not being able to participate included, visual problems, being unable to walk the length of hospital corridors, requiring ambulance transport to negotiate transfers and three flights of stairs with no lift, fatigue, particularly following stroke. Some service users who declined viewed research participation “for the general good”, as a low priority compared to personal “recovery” and keeping up with medical appointments.

Retention in the study was high, influenced by careful, facilitative and respectful planning and enactment by the academics. ARGs in the south met nine times (from November 2014 to June 2015) with approximately one month between meetings. Each session lasted for 1.5 hours. In the north, groups met for eight two-hour sessions (from October 2014 to May 2015). There were always two academic facilitators present, the academic research associate (RA) in each site and one or occasionally two academics who acted as co-facilitators. Patient and service user participation was consistent in both sites, occasionally a service user missed a session due to illness or a prior commitment but there was a minimum of four at each meeting.

Service provider attendance was more challenging. In the stroke rehabilitation setting there were consistently four or five staff members present for group meetings. In the dermatology outpatient setting, service pressures, shifts and annual leave frequently required staff members to be elsewhere, meaning they might arrive late or need to leave early, but a minimum of two at each meeting was achieved. Overall, commitment to the project was high in both sites. Attendance was good for both older adult participants (range 8–5 meetings, M = 7.2) and HCPs (range 7–5 meetings, M = 6). In addition, at the stroke rehabilitation site, where a total of 9 ARG meetings were held, older adult service user attendance was good (range 7–9 meetings, M = 8.2), as well as for HCPs, (range, 8–4 meetings, M = 7.3). One staff nurse withdrew from the southern ARG group following meeting 4, citing clinical demands. No other participants withdrew from either study sites ARGs. At the outset it was established that while there would need to be a commitment to attend all ARG meetings, there would be occasions when participants would not be able to attend, perhaps due to clinical demands or other personal commitments. Thus, there were no minimum number of sessions required for ARG membership, and we would not consider participants as having dropped-out of the study unless they stated they would no longer be attending the meetings.

**Figure 2.**
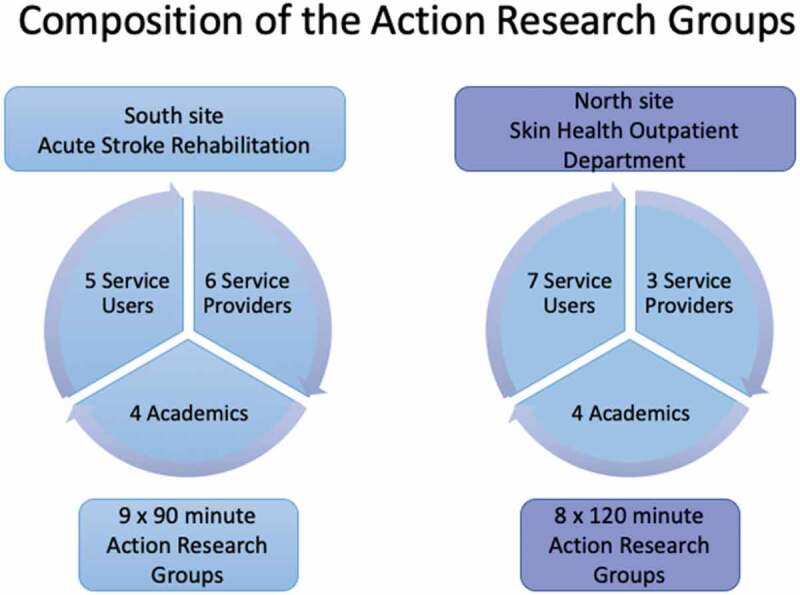
Composition of the tripartite Action Research Groups

Several patients and service users indicated their motivations for sustained participation that was core to project progress. These included, wanting to “do something for the community” and wanting to “help others” [who shared what they themselves had been through], to “give something back”. There were also expressions of interest in lifeworld perspectives in wanting to share with others what the experience of for example, psoriasis, skin cancer, hemiplegia or disruption in confidence was like. Most expressed an underlying desire for ongoing conversation with staff, wanting to ask questions about their condition and prognosis and give positive feedback including a desire to thank staff. Figure 2 below summarizes tri-partite action research groups.

### Stages of theory-led action research process

In the first stage of the study both groups, facilitated by researchers, learned about a new humanization theory and explored the eight humanizing dimensions (Todres et al., 2009). Introductory approaches were different in the two settings. In the dermatology outpatient service (North of England), the RA explicitly introduced each dimension, provided an example and then invited discussion about how they linked with personal experiences. Conversely, in the stroke rehabilitation service (South of England), the introductory approach was implicit, experiences were shared and then linked to the humanizing dimensions. This created new understandings and insights relevant to each setting that focused on humanly sensitive care. In the second stage, the group members carried out a humanized care assessment of the setting, drawing on each group member’s experience of care in their setting. This stage involved listening to, and collecting examples of, both humanizing and dehumanizing practices and then collectively deciding how to take a more enhance care practice focus on the human dimensions of care.

A “humanized care” improvement plan was initiated within each setting; this involved creating dissemination materials and engaging in developmental activities to both share and transfer the study experience of the group to others in the setting. An overarching Appreciative Inquiry (AI) approach guided the hands-on activity and group reflections (Ludema et al. (2006)). AI demands a conscious choice to collaboratively focus attention on what is well in the lives of individuals, groups and organizations and supports shared understanding (Lewis, 2016), this was a strong philosophical basis for guiding group facilitation. The theory-led action research approach took the form of an iterative spiralling through: (a) reflection on problem/theory/followed by gathering of descriptions of experiences, (b) actively sharing of perspectives, (c) facilitated use of finding good words to link together service user experiences with humanizing dimensions (theoretical ideas), (d) further reflection on problem/theory through service user perspectives to surface emerging solutions or new insights. The spiralling cycle offered an iterative practice improvement process (Mc Niff, 2020). Table II summarizes the focus, discussion topics and specific activities of each of the ARG meetings that underpinned this iterative spiral process. The credibility of the process can be judged by readers as in any action research process, i.e., making judgements about the action research credibility and trustworthiness through the fidelity of the account and its “fit for purpose” underpinned by transparency, use of reflection, participation, description of practical insights and transferability offered (Mc Niff, 2020). Table II summarizes the focus and specific activities of each of the ARG meetings that underpinned the practice improvement process.

**Table II.** Action Research Group (ARG) Sessions: Introducing the Humanizing Care theoretical framework and linking conceptual ideas to participants’ experiences

Dermatology outpatient service (North)	Stroke rehabilitation service (South)
**ARG 1** **Theory Engagement** Introductions to each other and discussion of the Humanizing Care Framework (HFW) as a large group. Research associate introduced the dimensions, provided an everyday example and then invited discussion from the group. Discussion covered: Agency, Uniqueness, Togetherness, Insiderness	**ARG 1** **Laying the foundation** Introductions to each other and the project, establishing a sense of group security, respect and togetherness. What makes us feel human. Sharing experiences of stroke care
**ARG 2** **Theory engagement through making links with participants experiences** Discussion of the HFW as a large group. Same format as group one meeting but additional examples of the dimensions were created using service users’ examples and stories of their experiences that had been gathered from previous week, ARG1. Dimensions covered were: Personal Journey and Sense making	**ARG 2** **Eliciting experiences of care following stroke** Sharing experiences of giving, receiving and researching stroke care through creating images with wool and stones. Discussing what these experiences look and feel like.
**ARG 3** **Theory engagement** Discussion of the HFW as a large group. Same format as ARG group two. Dimensions covered: Sense of place, Embodiment, Objectification and Reductionist Body	**ARG 3** **Theory engagement** Discussion of the eight humanizing dimensions with brief user-friendly description in two small groups. Participants respond to the framework and begin to review their understandings of the dimensions.
**ARG 4** **Theory engagement** Discussion of the HFW in a large group. Same format as ARG two and three. Dimensions covered: Passivity, Homogenization, Isolation, Dislocation, Loss of Meaning and Loss of Personal journey	**ARG 4** **Theory engagement** In two small groups with four dimensions per group, participants review their experiences of stroke care from groups one and two and link them to their dimensions. Participants evaluate the ease of matching experiences to one or more dimensions.
**ARG 5** **Humanizing self-assessment** Appreciative inquiry methods used to Identify what participants most value about the dermatology service. Identifying key areas: relationships between staff and service users, retaining specialist skills, staff who know personal history of skin conditions.	**ARG 5** **Humanizing self-assessment** Appreciative inquiry methods used to identify what participants most value on the stroke unit and Early Supported Discharge service. Identifying key areas: staff-service user relationships, a kind and welcoming culture, gentle, ongoing explanations.
**ARG 6** **Humanizing self-assessment/improvement plan** Drawing examples from the “what we value” discussion into the Humanizing Framework, pointing to links and assessing how the groups could continue a focus on humanizing care.	**ARG 6** **Humanizing self-assessment/improvement plan** Review of what works well and the tensions inherent it keeping a human-centred focus within service-centred health care settings. Narrowing down from what’s valued to potential areas of interest for a small service improvement
**ARG 7** **Humanizing Improvement Plan** Review of themes from previous session. Narrowing down and prioritizing the humanizing interventions.	**ARG 7** **Humanizing Improvement Plan** Action planning small service improvement initiatives around raised awareness of the human aspects of care.
**ARG 8** **Humanizing Improvement Plan** Finalizing potential humanizing interventions e.g., the “huddle” to share specialist skills and knowledge; a board documenting examples of humanizing care. Concluding group and agreeing the format of disseminating findings to the unit and hospital staff. Reflection on the ARG process and experience and wider dissemination.	**ARG 8** **Humanizing Improvement Plan** Identifying ways to keep humanizing care alive on the unit and spread to other areas of care e.g., planning production of a DVD of humanizing care stories to share in training sessions and a ward-based humanizing care tree to raise awareness of “humanizing moments.”
**Group arranged a meeting with Trust service managers and staff to share the ARG outcomes as wider practice impact**. Service users presented some of their experiences as linked to the theory as part of the practice impact outcome.	**ARG 9** **Conclusions and knowledge for practice** Finalizing plans for producing a digital film clip and humanization tree. Discussion of dissemination meetings and events. Group activity to develop images of humanizing stroke care. Reflection on the ARG process and experience.

### Data analysis

Data sources, analysis, and purpose of each activity to underpin both “within setting” and “across setting analysis” are summarized in Table III. All group meetings were audio-recorded, transcribed verbatim and anonymized. Data were reviewed reflectively, clustering examples of experiences relating to each of the eight dimensions of humanizing and dehumanizing care, and then reflecting on the meaning. This was not a thematic or content analysis in the conventional sense, but rather was sensitized by a process of reflecting on meaning in the data and clustering of meanings (not dissimilar to a Reflective Lifeworld Research stance after Dahlberg et al.,) but one which was attuned to the dimensions of the humanizing care theory as a background. This entailed a reflective back and forth process between the data, the theory and the meaning of the experience in “feeling human” or otherwise, with further reflection on the relevance to a dimension of the theory. In reviewing the transcripts, the research team also made analytic notes and reflected on group activities, group process and dynamics and responses to the activities, as a way of evaluating what worked in the facilitation process. Overall attention was given to the application of the humanization themes and characteristics of lifeworld-led facilitation that seemed to work well or otherwise and findings related to what facilitated engagement and any group difficulties encountered. Therefore, activities that worked particularly well and challenges encountered were explored and documented as part of the in-depth reflective analysis.

**Table III.** Summary of data sources, “within setting” and “across setting” analysis

**Level one analysis – within setting**			
	**Data source**	**Data analysis**	**In order to**
A	Transcripts of meetings	Were reviewed and analysed qualitatively to identify what experiences were described by • older people • staff as humanising or dehumanising	Investigate what healthcare experiences and practices are important to older people in making them feel human
B	Reflections of research team	Explored to identify how easy/difficult it was to consider the humanisation framework (HFW) together	Discover how easy/difficult it was to introduce and explore together a new, conceptual framework based on humanisation theory to service users and service providers
C	Group notes	Were used to assess and identify a) how people decided what to do b) what supported this activity	Identify the human aspects of care and practice that could be developed in both settings within a targeted ‘quality improvement initiative’ led by new theory
D	Group notes /reflection	Were used to a) describe what happened re plans, implementation and outcome b) describe what needs to be in place for this to happen	Plan, implement and assess a humanising services improvement process in each site Evaluate the impacts and outcomes of the action research process in each site
**Level 2 analysis across setting**			
	Comparative analysis of B, C and D	To highlight similarities and differences in the two research settings, offering a comparative analysis to add context to the findings	
	Comparative analysis of B, C and D	To identify transferable processes that have potential to enhance dignity in care for older people in other human service areas	
**Purposive activity to enhance transferability**			
	Humanisation Toolkit/ Guidebook and digital film (Pound et al., 2016)	To produce transferable strategy materials	Share our understandings of ‘what works’ in humanising service with other practitioners

Recordings of the ARGs were transcribed and data reviewed and analysed in an iterative process that allowed the research team to understand how people conceptualized humanization and to identify next steps to be taken. This process also enabled identification of how well, and in what way, experiences related to the eight dimensions of the humanization framework. Key experiences that patients and service users highlighted as having a significant impact upon them were analysed using the humanizing care framework as a sensitizing background. For example, they were asked to describe important moments of humanly sensitive care, or otherwise, concerns or important turning points within their healthcare journeys to help illuminate the human aspects of practice under discussion. Data concerning all aspects of the decision-making process about what really matters in relation to human aspects of care and practice and ways to make services more humanized were discussed and documented in each meeting. These data were subjected to reflective analysis to assess the ease and relevance by which the humanizing conceptual framework could be translated into useful directions for “humanizing practice”. A comparative analysis of data across the two settings was also of particular importance in delineating transferable aspects of the humanizing improvement strategy. Table III provides an overview of sources of data and the purpose of the analysis process.

## Findings

### Understanding the meaning and relevance of the theoretical framework

Over the course of ARG meetings, we did not experience any insurmountable barriers to the groups fully engaging with the humanized care theoretical framework. While initially one group experienced some difficulty in grasping theoretical details and language, once theory was specifically linked to examples of individual experiences to assess what each of the humanizing dimensions meant to each individual group member, understandings were shared and deepened by all group members (as early as Action Research Group meeting 2). This indicated practical utility of a lifeworld-led approach, whereby everyday experiences shared by service users revealed deeper aspects of how human or otherwise the experience felt, and this was in a participative sharing context.

**Figure 3.**
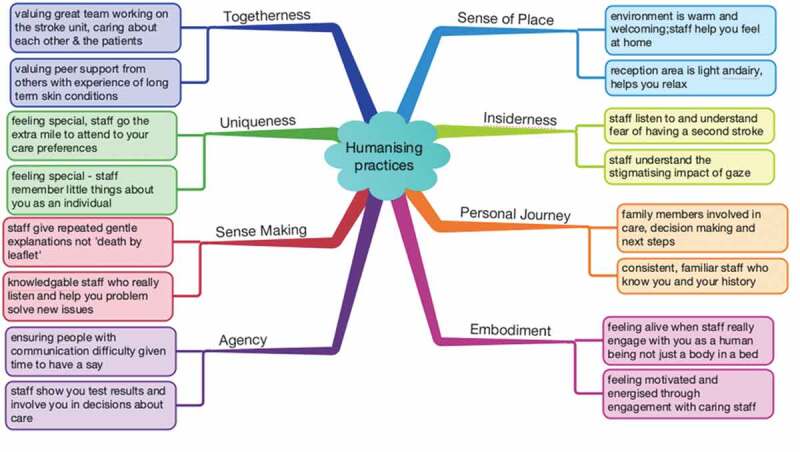
Examples of humanizing practices that older people from both settings identified

Common to both settings, participants valued space to listen to shared lifeworld experiences, engaged in group reflection about examples of the human dimensions of care underpinned by personal experiences and provided resources for meaningful discussion of the implications in each setting. All participants expressed that they were emotionally moved by listening to others’ experiences, were able to link examples of experiences to each of the theoretical humanizing dimensions and expressed that they were collectively passionate about a focus on humanly sensitive aspects of care in the specific setting. As anticipated, using a lifeworld experience approach was powerful in bringing the dimensions “alive” in each setting. The dimensions ARGs readily engaged with early in the process included; sense-making, sense of place, personal journey. Those worked through more slowly and which were experienced as more complex and needing greater reflection included embodiment, insiderness, uniqueness and agency. Although the groups used an AI lens to foreground good practice, inevitably some stories and experiences were readily associated with experiences and understandings of what can make care a dehumanizing experience emerged and these were vitally important in clarifying each dimension with a continuum of examples negative and positive. Figure 3 illustrates some examples of practices from both settings that patients and service users pointed to as humanizing, as led by each of the theoretical dimensions, and in participants own words.

In addition to providing concrete examples of humanized care, service users valued the little things for example, demonstrating an understanding of what it was like for the person, even if the situation could not be changed; a smile; a warm introduction on first meeting; clear gentle explanations, and a demonstration by the practitioners that they understood the difficulties encountered by the person and could navigate implications of professional concerns such as service targets. There are a number of setting specific findings which include: Increased appreciation of the impacts of stroke and the challenges to stroke care providers of meeting each service user in a way that remembers and cares about humanly sensitive approaches in care; increased understandings of what it is like to live with a long-term skin condition and the importance of holistic specialist skills to support older people in this situation; increased skills in working in a lifeworld-led attuned mode and increased insights into the value of using and trusting this kind of sensitization and learning as part of a rigorous and novel ARG process.

### The process of engaging with the humanizing framework

Extracts of discussions which illustrate how ARG members responded to the application of theoretical framework to assess humanizing aspects of practice are offered in Table IV. The explicit and implicit strategies refer to different ways of learning about humanizing theory. In the explicit strategy the framework was introduced to ARG members as a conceptual framework, followed by inviting examples from personal experiences. The implicit strategy involved prioritizing service users’ experiences and stories of care and only later aligning these with the humanizing dimensions. We were particularly interested in testing out if the humanizing theory could “be applied”, in a conventional sense, that is, could we facilitate a group of service users and practice professionals to work through the theory in an explicit way? We wanted to assess what would happen. In contrast, we wanted to compare how such a conceptually driven approach worked with a facilitation approach that was more emergent by implicitly using the theoretical framework, so that the theory remained in the background and where the natural group process led by a range of facilitation activities was forefront.

**Table IV.** Transferable learning: The value of engaging with the theoretical framework for humanizing care framework

Meaning and Transferable Learning	Dermatology outpatients (north) Using an explicit Strategy	Stroke rehabilitation (south): Using an Implicit Strategy
**Listening to lifeworld examples from stories was moving and deepened understandings. It was helpful to service users and staff**. **Sharing service user experiences gave opportunity for staff to reflect on what it was like for older people, an inside view, and this was in contrast to the professional more external organizational view.**	*Different ways of listening (Staff)* *This could be dry—but [listening to experiences] have made it come alive (Service User)* *Because sometimes I find when patients aren’t happy about their care, it’s not necessarily about the diagnosis, it’s about the way they were treated, sometimes it’s those aspects of care that the patients aren’t happy about and that’s the human side … and I think that’s what you’re trying to put in in’ humanizing.’(Staff)*	*That’s what stood out for me. Usually you have a group where you discuss things and it’s just … it’s just nursing staff and therapists and professional staff and and you don’t see it from the patient’s point of view. (Staff)* *What stood out for me was having the nurses from the wards to hear their opinions as well as all of ours as well, that was really good and interesting for me. … And I found that helpful because you understand from the other side. (Service User)* *I like the discovering what … especially like with the patients, what their experience was like, because you don’t know that, you just … it’s something new that you don’t know (Staff)*
**Time, space to listen, to talk honestly about inner lifeworld perspectives rather than a more external view of goal setting, unit processes, physical outcomes was helpful.**	*… people do find it more difficult, so I was quite prepared, even though I wasn’t sure which way we were going, to give it time and see. And yet in discussing it into different categories, yeah, it was OK (Service User)* *… found that helpful because you understand from the other side (Staff)*	*It’s like therapy (SU)* *… reflecting from last time, answering to Betty to say it did feel really good to sort of sit down and [hear experiences] and that felt almost therapeutic. (Staff)* *just the time to reflect and the time to think about making things better, just the opportunity for that! (Staff)* *Great way to get people to think about and express their experience, and definitely a lot that I will take forward for a long time (Staff).*
**The language of the theory was perceived as difficult at times but became clearer through using experiences**. **A process of gathering a range of words to express each dimension was a helpful reflection.**	*So some of these have a reason and they can bring it out—but I didn’t know what they called them (the humanizing dimensions), obviously, you’ve got a name for them but we didn’t have a name for them (Service User)* *… you had to revisit some of them over again, though, because it was almost as though we got to learn what the dimensions were, your experiences, where those experiences fit into those dimensions, so they kept coming up a lot (Service User)*	*We just said that reading these by themselves I thought, oh, I don’t have a clue what they’re going on but when we’ve had them and putting them [the stories and experiences] in, it seemed to make more sense (Staff)* *there are these dimensions that exist to define humanizing care, and then we’ve kind of put it in our own words (Staff)* *I think the humanizing framework was useful in putting it into words why that would be humanizing or dehumanizing*, *[then thinking through in our own words and language] helped to think about all the different reasons why something could be humanizing or dehumanizing (Staff)*
**Understanding the nature of the theoretical framework made sense to service users and health care staff although it took time and needed a facilitated process**. **The continuum of dimensions and humanizing framework terminology helped groups reflect on what that dimension might look like in practice/in everyday life**. **The application of the framework helped ARG members get in touch with their core values and this was welcomed.**	*It has been useful, particularly to get the human side of care over, it’s almost as though you’re putting values into headings that people can relate to and what a difference that has on somebody else. Because I was once told the smallest action you can do in a day can either make or break somebody’s day; you know, a crossed word with somebody or you can upset that person (Staff)* *HFW is deep and complex and this is appropriate because life is complex- need something that has a depth—but need to transfer it into something meaningful without making it meaning less (RA).*	*The branches of people who need a big heart, all the people that deal with all the branches of other’s stroke, you know, the therapies, the speech therapist, all that … And that’s humanizing care, having the big heart to deal with it. (Service User)* *I think it’s nice having it on a continuum because as we’ve had in this discussion, some people want to be unique/don’t want to be unique, want to be alone/don’t want to be alone so to be able to place yourself somewhere on some of those is quite useful, rather than doing it binary (Staff, Service User added agreement)* *Yeah I think it would be interesting to think about it some more. But I think it does cover everything (staff)* *When C was saying earlier about humanizing care champions, I was thinking I think that’s something that we probably do already do a little bit of but I think we could acknowledge a lot more in terms of when someone’s done something that’s really humanizing (staff)*

In both settings adoption of an appreciative approach was powerful in helping the groups and individuals feel safe to consider situations which were previously considered “problems” and potentially avoided, particularly examples of dehumanizing moments or situations in practice Additionally motivating factors that were important to both service users and to health care staff emerged that served to sustain interest in ongoing participation in the ARG’s Staff reported that they found the learning about human dimensions of care alongside a participatory action research approach attractive as it offered opportunity to work with and alongside patients in making a difference to care. This can be captured as an interest in participating in “something a little different”, group tasks relevant to human dimensions of care, and feedback from participants that flags the value placed on the collaborative nature of applying the framework. Purposively designed group activities worked as a way to really listen to what each person did, and what they experienced, for example, participants created a typical day in their life “on the unit”. Both service users and care providers participated together to explain what it was like for them. Such was the interest in the processes and opportunities of the project that several staff reorganized work shifts to attend or participated during their days off, generously helping overcome a potential “shortage of time” barrier presented to the project team. Several staff were very engaged with and attracted to the “being human” theme and all staff, were keen to engage with service users as highlighted in the extracts of data in Table IV.

### Transferable learning across the two sites

Analysis of the data revealed a number of commonalities in how service users and service providers experienced the content and process of being introduced to and interacting in experiential and theoretical ways with the humanizing framework. There didn’t seem to be any distinct differences in whether an explicit or implicit strategy was used, both groups successfully worked through the facilitated process, whether conceptually driven or otherwise. What seemed to be transferable to both settings, and what emerged as practically useful was the use of lifeworld stories, the quality of the reflective space and the guiding role of the humanizing theory whether conceptually explicit to the group or implicit. Data from staff and service users presented in Table IV illustrates the transferable learning that emerged from each setting and which can inform a pathway to potential practice impacts. For the purposes of succinctness, the findings are summarized as a table to illustrate similarities that emerged, even though contrasting implicit and explicit strategy was used, the insights from each group did not contrast. Data from staff and service users presented in Table IV illustrates the transferable learning revealed by the comparative analysis.

Findings from the comparative analysis point to the benefits of helping teams reconnect with humanizing care values and harnessing the energizing properties of this in collaboration with service users, so this is a taking a step back to look again at what is important in the context of what matters to the older people engaged in the process within two distinctive settings. Several areas emerged which offer transferable learning for practice improvement strategies which include: Listening to lifeworld stories sensitized by humanizing dimensions; sharing of user experiences and reflections on what happened to them, both positive and negative; creation of time and space to listen and share to inner world perspectives; using lifeworld experiences to make links with humanizing theory dimensions and language; gathering of words from experiences that worked for the group in shared understanding alongside application of the framework through thinking about everyday happenings that was central to the facilitation of the groups. A human dimension informed care focus was experienced as valuable and practical, both in an explicit and implicit theory application process. The findings illustrate how a meaningful step forward in-service improvement can be achieved by combining a distinctive focus on forms of humanization and forms of dehumanization given by the theoretical framework and which is informed by patients own experiences and journeys in each setting. This rich lifeworld evidence is useful in specific settings of dermatology and stroke rehabilitation but also reveals transferable processes that have potential to enhance care in impactful ways for older people in other human service areas.

## Discussion

The transferable strategies concern firstly how application of the theory underpinning participatory processes was implemented and emerged as a distinctive life world-led process. Second, effective ARG processes and strategies to overcome challenges that were encountered are useful learning. The impacts and outcomes of the project have contributed to resources that have been designed to lead and support care teams wanting to engage in a humanizing care improvement project in the future. In the context of this humanizing care improvement project, we discussed and developed activities, techniques, and facilitation styles which are consistent with a lifeworld-led approach. Transferable features of the facilitation approach, which we argue can enhance meaningful practice impacts, include the following characteristics as summarized in Table V.

**Table V.** Characteristics of a lifeworld-led facilitation approach

Establishing lifeworld- led conditions	Attending to lifeworld- led activities	Challenges and transferable learning
Using a room and surroundings where people felt comfortable and safe and where experiences were valued, not judged. Striving to keep the atmosphere and tone relaxed and friendly by using humour, warm greetings, and not rushing goodbyes. Making sure people know what was happening and what is expected (summarizing, a clear but fluid agenda that prioritizes their experiences). Keeping to time but avoiding rushing (planning time allocations in advance). Fostering a sense of respect and tripartite group equality (ground rules and facilitation to support equal opportunity to hold the floor and demonstrate personal experience/expertise). Creating a sense of trust through tone and gentle explanations and identification of humanized care practices and when group feel secure potentially dehumanizing practices. An Appreciative inquiry methods approach can create optimum conditions for this.	Engaging in activities which encourage equality, involvement and participation. Reflecting upon, being aware of and keeping in check professional or medicalized perspectives as discussion of experiences emerges. Choosing activities which reflect creative and embodied ways of knowing and participation rather than relying entirely on verbal description, patient “reports” or feedback and organizational explanation (E.g., use of coloured stones and wool to represent experiences and help keep discussion open ended and not pre-determined.) Encouraging maximum participation and collaborative listening and storytelling by organizing into smaller groups and thinking about best ways to subdivide groups that will foster dialogue about older peoples’ experiences. Introducing images (e.g., in card task) which represent lifeworld domains e.g., natural world, nature, connectedness, social relations, time, mood, people and the environment. Encouraging fun, creativity, exploration and a sense of freedom without knowing where it will take the group. Being courageous and honest e.g. raising negative issues witnessed in the service and emotional reactions to them, responding to older peoples’ experiences whether positive or negative. Modelling an open, receptive and interested way of being Joint, equal decision-making as groups progress, particularly in planning service improvement phase Checking in regularly with the groups between meetings to see how things are going for them.	Sometimes reliance upon service providers to facilitate small groups, could result in discussion becoming more medically/professionally led than service user led Investing time to build relationships, trust and confidence so that participants are not overly sensitive to negative comments but able to embrace what different experiences mean in humanized care terms. Uncertainty is inherent in the process, this has potential to create a confusing sense of “not knowing” and therefore needs ongoing clear description of how *the process* will develop over the coming weeks As with any group facilitation managing more dominant or talkative members of the group Facilitators require skills and experience of facilitation—e.g., being very comfortable with a process that is more organic and uncertain, rather than a more structured, controlling focus on aims and outcomes. Holding “one’s nerve” when introducing new and potentially unusual activities. Being prepared for emotional reaction and being skilled/confident in managing “pivotal moments”. Teasing apart what is lifeworld-led facilitation (a focus on lifeworld experiences and what they mean in humanizing or dehumanizing terms) and what is good group facilitation e.g., creating conditions for service users to share their experiences and for service providers to reflect upon them. Readiness in the setting/system Preparatory work to ensure teams are open to/want to explore humanized care ideas/value lifeworld evidence.

In our experience a key characteristic of facilitators in this kind of theory-application-to- practice initiative included confidence in the theoretical framework with understanding of its aims and ability 'to weather' the uncertainty of others. Therefore, it is important to attract people to participate who have a desire to explore new practice improvement ideas, to adequately prepare them for facilitation and also to provide tailored resources for facilitation (we have devised a toolkit and film for this purpose (Pound, 2016). Each of the experimental ARGs engaged in the following rigorous steps: Theory engagement: Introduction to the humanizing dimensions, with a focus on positive humanizing examples first, then moving onto negative dehumanizing examples as the group were ready. Discussion was encouraged that was lifeworld led, taking a core focus on service users experiences in dermatology or stoke rehabilitation relevant to the humanizing dimensions. Through this focus on experience, what matters to older people in any setting can be explored and a humanizing context for future discussion can be set. In addition, this theory engagement process allowed a type of 'humanizing self-assessment' for the teams to reflect upon and facilitated the development of a Humanizing Improvement Plan with ongoing discussion of the Humanizing Improvement Plan and facilitation of actions that have been identified. As such, the study offers two impactful examples of application of the human dimensions of care framework in practice. Because the theory is embedded in a lifeworld- led care philosophy (Horberg et al., 2019; Todres et al., 2007), grounding discussions in personal experiences and stories was a practical and potent way to link individual experiences of receiving and providing humanly sensitive care to the human dimensions of the theory. A valuing of all kinds of knowledge by the participants emerged with an honouring of different personal experiences and different kinds of expertise rather than a privileging of technical or professional knowledge alone.

The theory-led nature of the ARG discussions allowed a keeping of humanizing dimensions in mind without having to “overpower” or distract attention away from the experiences. This was a kind of back and forth movement between experiences and theoretical dimensions. Here, experientially grounded examples were vital to illustrate what each of the humanizing dimensions pointed towards. If the definition of a humanizing dimension was “read out”, the group were perplexed, but the examples quickly aided understanding and helped groups to work beyond the theoretical language and to apply the ideas to their own “experience near” examples. Using the Humanizing Framework as a scaffold for discussion, attuned to theory, in our experience facilitated a richer description of life world experiences at the human dimension level, this was a 'step change', rather than the more common focus of a general discussion on experiences of care.

A lifeworld perspective with participants’ everyday experience, was therefore a coherent and useful starting point for the research. It allowed ARGs to develop deep understandings of the issues at hand and may have helped group cohesion, as evidenced by no attrition in the sample of patients and service users or staff (Galvin et al., 2016). Our original approach is allied with similar moves to lead care that begin in the patient’s lifeworld such as Carel (2011) and her development of a phenomenological toolkit for use in medicine; the work of Ellis-Hill et al. (2019) in arts informed interventions in stroke rehabilitation; dialogical phenomenological approaches as advocated by Halling et al. (1994) and a growing body of work about patient perspectives on diagnostic categories (Weiste et al., 2018). We argue that provision of actionable pathways to enhance care that begin with patient experience and which are sensitized by humanizing dimensions of care theory are significantly impactful.

The theoretical framework also has potential to reconnect practitioners to the values that motivated them towards caring work, and which sustain their capacity to care. Therefore, our participatory project contributes new experientially rich understandings alongside a transferable strategy for the implementation of a more humanly sensitive approach to healthcare. We suggest this can contribute to deepening meaningful patient-led care (see further allied discussion in Dahlberg et al., 2009; Todres et al., 2014). Further, the approach reported in this present paper has potential to offer practical directions that are transferable to a diverse range of settings that wish to pursue meaningful person-centred care.

### Study strengths and limitations

Our key strengths are, firstly, the sustained engagement of two ARGs comprising older patients, service users, service providers and academics. Secondly, a distinctive lifeworld informed decision-making process that was led by the eight dimensions of the humanizing framework and informed by patients’ own journeys and experiences. Because the work has its foundation in phenomenological philosophy, the project’s characteristics allowed a focus on “a way of being” with older people, rather than a “doing more” and this minimizes “new initiatives overload” and made it easier for staff to consider in their practice. We have been have taken steps to sustain discussions about humanizing care that are reported elsewhere, see for example, Royal Bournemouth NHS Trust Humanising Care Project.

As in any action research project, learning has informed some transferable strategies to negotiate and overcome methodological issues. These methodological challenges include: Finding ways to increase the diversity of older people involved, which includes, for example, older people with severe and lasting impairments, those who have experienced difficult transitions, such as hospital discharges to care homes, and a range of family issues. Experiences of care might be quite different than those of the more able, who are in recovery or who are in remission from a long-term condition. As might be anticipated in the context of service pressures, direct involvement of senior staff is an ongoing challenge. Our reflections underlined the importance of a range of staff participating, front line staff to maintain humanizing work and senior staff/organizational support to validate it.

The decision-making process within the ARGs was unproblematic but when our findings were shared with a wider staff base, in one of the sites, some staff members raised objections stating “we do that anyway”. This has potential to give the project work a low value within such working culture, but also highlights the importance of gathering evidence of the need for humanizing care through using examples of dehumanizing care from service users’ lifeworld examples. If this is difficult and sensitive a further strategy would be to use lifeworld evidence from published studies relevant to the practice area. Further, the study demonstrates that an experimental action research approach can foster productive participation with meaningful collaboration.

## Conclusion

We have aimed to show how, by using a new phenomenologically informed framework for humanizing care, “what matters to older people” can be illuminated and acted upon in impactful ways. Further we offer transferable knowledge and a tested strategy for leading humanizing service improvements in other settings (Pound, 2016). A rigorous theory-led action research approach, with engagement of a tripartite teams of service users, health care staff and academics, not only enhances lifeworld led understandings of care, as led by everyday experiences of participants within each care setting, but crucially moves qualitative research findings to a second step: A philosophically informed approach to the core dimensions of what it means to be human can be applied robustly in transferable ways for enhanced health care improvements that are lifeworld led and grounded in meaningful patient experience. Given the characteristics of each setting, it is evident from our project that an action research process, led by humanized care theory, can be sustained over several months in busy service settings, with high turnover inpatient or outpatient services. Further, we have found that that both health care staff and service users valued their prolonged engagement in the process.

Variation in group ARG processes allowed us to test out ways in which the humanizing theory could be explored with tripartite groups and illustrates how service users and professionals were able to engage with philosophically grounded theory. An “implicit process” beginning in patient experience to translate humanizing theory is effective, embedding insights within everyday practice and this lends itself to a diverse range of groups and settings. An explicit strategy, beginning in understanding the theory, and then gathering examples from practice in participation with patients and service users is also useful and particularly where there may be a desire for more structure in the ARG sessions where there is limited time or limited facilitation resources. Lifeworld-led action research processes therefore have potential to offer significant impacts in practice in partnership with service user and patients in a diverse range of settings and offer a way to deepen person-centred approaches to care. Such approaches, informed by strong theoretical foundations that attend to meaningful experiences can *do justice* to the complexities of human life within a care context and can contribute to meaningful person-centred care by offering alternative descriptive power to the medical model and social models, of for example, disability. Here a lifeworld-led approach can mediate oversimplifications in patient-—led care such as “more choice” and at the same time facilitate a particular kind of participation. Directions for practice development can emerge directly from people sharing their experiences sensitized by phenomenological oriented theory in an action research context.
